# Exploring transcriptional signalling mediated by OsWRKY13, a potential regulator of multiple physiological processes in rice

**DOI:** 10.1186/1471-2229-9-74

**Published:** 2009-06-18

**Authors:** Deyun Qiu, Jun Xiao, Weibo Xie, Hongtao Cheng, Xianghua Li, Shiping Wang

**Affiliations:** 1 National Key Laboratory of Crop Genetic Improvement, National Center of Plant Gene Research (Wuhan), Huazhong Agricultural University, Wuhan 430070, PR China

## Abstract

**Background:**

Rice transcription regulator OsWRKY13 influences the functioning of more than 500 genes in multiple signalling pathways, with roles in disease resistance, redox homeostasis, abiotic stress responses, and development.

**Results:**

To determine the putative transcriptional regulation mechanism of OsWRKY13, the putative *cis*-acting elements of OsWRKY13-influenced genes were analyzed using the whole genome expression profiling of *OsWRKY13*-activated plants generated with the Affymetrix GeneChip Rice Genome Array. At least 39 transcription factor genes were influenced by OsWRKY13, and 30 of them were downregulated. The promoters of OsWRKY13-upregulated genes were overrepresented with W-boxes for WRKY protein binding, whereas the promoters of OsWRKY13-downregulated genes were enriched with *cis*-elements putatively for binding of MYB and AP2/EREBP types of transcription factors. Consistent with the distinctive distribution of these *cis*-elements in up- and downregulated genes, nine WRKY genes were influenced by OsWRKY13 and the promoters of five of them were bound by OsWRKY13 *in vitro*; all seven differentially expressed AP2/EREBP genes and six of the seven differentially expressed MYB genes were suppressed by in *OsWRKY13*-activated plants. A subset of OsWRKY13-influenced WRKY genes were involved in host-pathogen interactions.

**Conclusion:**

These results suggest that OsWRKY13-mediated signalling pathways are partitioned by different transcription factors. WRKY proteins may play important roles in the monitoring of OsWRKY13-upregulated genes and genes involved in pathogen-induced defence responses, whereas MYB and AP2/EREBP proteins may contribute most to the control of OsWRKY13-downregulated genes.

## Background

WRKY genes, which encode proteins binding to the *cis*-acting element W-box, have been isolated from many plant species [1,2]. During the past decade, numerous reports have indicated that WRKY genes are involved in defence responses (*Arabidopsis AtWRKY6*, [3]; *AtWRKY18*, [4]; *AtWRKY70*, [5]; *AtWRKY33*, [6]; and rice *OsWRKY03*, [7]; *OsWRKY71*, [8]; *OsWRKY13*, [9]; *OsWRKY45*, [10]), development (*TRANSPARENT TESTA GLABRA2*, [11]; *MINI3*, [12]), hormone regulation (*OsWRKY51 *and *OsWRKY71*, [13,14]), as well as sugar signalling and sesquiterpene and benzylisoquinoline alkaloid biosynthesis (*SUSIBA2*, [15]; *GaWRKY1*, [16]; *CjWRKY1*, [17]).

The most stringent definition for a WRKY binding site, a W-box, is a hexamer of TTGAC(C/T), which is found in the promoter regions of many pathogenesis-related genes [18]. Based on the core sequence (TTGAC) of a W-box, there are variant W-boxes, TTTGACA, TTTGAC(C/T), TTGACTT, TTGAC(A/C), TTGAC(A/C)A, and TTGAC(A/C)(C/G/T), and a W-box like *cis*-element, TGAC(C/T) [18-21]. Recently, another variant W-box, TTGACG, which carried a minimum *cis*-element *as-1 *(TGACG) for the TGA transcription factor, was reported to be bound by rice OsWRKY13 transcription factor *in vitro *[9]. Furthermore, another novel WRKY binding site PRE4 (TACTGCGCTTAGT), which was identified in the promoter of *OsWRKY13*, participates in the self-regulation of *OsWRKY13 *[22]. Previously, barley WRKY protein SUSIBA2 was reported to specifically bind to the sugar responsive *cis*-element (SURE) in addition to a W-box [15]. Tobacco NtWRKY12 can bind to a WK-box (TTTTCCAC) in the *PR-1a *promoter, which deviated significantly from the consensus sequence of a W-box [23]. These results suggest that the *cis*-elements for the action of WRKY proteins are variable.

Computational methods that define relationships between gene expression levels and putative regulatory sequences in the promoter regions of differentially expressed genes based on large-scale microarray data and genome sequence screening are increasingly being used to establish a signal transduction network [18,24,25]. Evidence from microarray studies revealed an overrepresentation of W-box elements within the promoters of a cluster of genes that are coexpressed during systemic acquired resistance [18]. Transgenic AtWRKY70 microarray experiments showed that W-box elements are similarly enriched in both up- and downregulated clusters predicted by a bootstrapping program [20]. Thus, the potential relationship between different genes, including WRKY genes, may be obtained by integrating the knowledge of WRKY or other transcription factors and their related regulatory elements.

Rice OsWRKY13 is a potentially important transcriptional regulator involved in multiple physiological processes. It mediates disease resistance to bacterial blight caused by *Xanthomonas oryzae *pv. *oryzae *(*Xoo*) and fungal blast caused by *Magnaporthe grisea *through activation of salicylic acid (SA)-dependent pathways and suppression of jasmonic acid (JA)-dependent pathways; OsWRKY13 can bind to the W-box and W-box like *cis*-elements that are present in the promoters of some pathogen-induced defence-responsive genes [9,22]. Furthermore, genomewide analyses of the expression profiling of *OsWRKY13*-activated lines reveal that OsWRKY13 directly or indirectly regulates the expression of more than 500 genes [26]. OsWRKY13 is also a potential regulator of other physiological processes during pathogen infection. It activates redox homeostasis by the glutathione/glutaredoxin system as well as the flavonoid biosynthesis pathway, which may enhance the biosynthesis of antimicrobial flavonoid phytoalexins [26]. OsWRKY13 inhibits the SNAC1-mediated abiotic stress defence pathway and terpenoid metabolism pathway to suppress salt and cold defence responses as well as to putatively retard rice growth and development [26]. Compared to the large number of differentially expressed genes in *OsWRKY13*-activated plants, however, most OsWRKY13-regulated pathways have yet to be elucidated.

To understand the transcriptional regulation of OsWRKY13, the types of transcription factors and conserved motifs in the promoter regions of the genes differentially expressed in *OsWRKY13*-activated plants were analyzed. The results suggest that the actions of OsWRKY13 on the expression of more than 500 genes are partitioned by different types of transcription factors through binding to distinctly distributed *cis*-acting elements in the promoters of OsWRKY13-upregulated and -downregulated genes. Furthermore, OsWRKY13 appears to bind preferentially to the promoters of downregulated genes *in vitro*, suggesting that it may function more as a negative transcriptional regulator.

## Methods

### Microarray data

The microarray data, generated using Affymetrix GeneChip Rice Genome Arrays, were from our previous report [26] and the data were released under accession number GSE8380 of the Gene Expression Omnibus (GEO) database http://www.ncbi.nlm.nih.gov/geo. The data were generated from the leaves of a pool of 20 4-week-old wild-type Mudanjiang 8 (*Oryza sativa *ssp. *japonica*) plants and *OsWRKY13*-overexpressing independent homozygous transgenic lines, D11UM1-1 and D11UM7-2 [9]. D11UM1-1 and D11UM7-1 carry two and one copies of the transgene, respectively, and the two lines have more than 20-fold higher *OsWRKY13 *transcript levels than wild type with or without pathogen infection [26].

### Promoter analysis

The rice genomic sequence was obtained from TIGR (The Institute for Genomic Research, http://rice.tigr.org) Rice Genome Annotation version 4.0. The 2-kb sequence upstream of the known or predicted coding region of rice genes that are differentially expressed on the microarray chip were identified with a 'present' call using the MAS5 method (version 5 edition, Affymetrix, Inc.) and their annotation was extracted. In total, 18,362 promoter sequences were filtered for further analysis. To search for overrepresented motifs within the promoter sequences of these genes, we performed one modified Perl script according to the enumerate methods of one- through 10-mer in the coregulated set of promoters (Sift program; [27]). The number of occurrences of each motif was compared with an expected value derived from the frequency of that element in the whole microarray (18,362 promoter sequences as baseline control). The overrepresented motifs in up- and downregulated genes were confirmed using the binomial distribution [27]. Only the motif with *P *value < 1e-5 (e = 10, 1e-5 = 1 × 10^-5^) was considered significant and selected for further analysis. Comparison of the detected overrepresented motifs with known *cis*-elements was performed using the PLACE http://www.dna.affrc.go.jp/PLACE/signalscan.html[28] and PlantCARE http://bioinformatics.psb.ugent.be/webtools/plantcare/html[29] databases and literature searches.

### Rice transformation

To construct an RNA interference (RNAi) vector of *OsWRKY13*, a 900 bp cDNA fragment of *OsWRKY13*, obtained by PCR amplification from *OsWRKY13 *cDNA clone EI12I1 [GenBank: BF108309] [9] using primers WRKY12F (5'-**GGGGACAAGTTTGTACAAAAAAGCAGGCT**GTGATGGCGGCAGGAGAG-3') contained attB1 site (in bold) and WRKY12R (5'-**GGGGACCACTTTGTACAAGAAAGCTGGGT**TGAACACGACGGCGCACTC-3') contained attB2 site (in bold), was inserted into the pHELLSGATE2 vector by BP and LR reactions (Gateway Kit, Invitrogen, USA). *Agrobacterium*-mediated transformation was performed using calli derived from mature embryos of rice variety Minghui 63 (*O. sativa *ssp. *indica*) according to the protocol of Lin and Zhang [30]

### Pathogen inoculation

Plants were inoculated with *Xoo *strain PXO61 at the booting stage by the leaf clipping method [31]. Rice variety Mudanjiang 8 was susceptible to PXO61 and variety Minghui 63 (*O. sativa *ssp. *indica*) was moderately resistant to PXO61. Mock-inoculated (control) plants were treated under the same condition except that the pathogen suspension was replaced with water.

### Quantitative reverse transcription-PCR

For RNA isolation, 5- to 6-cm leaf segments located below the inoculation cutting sites were obtained. The RNA sample for *OsWRKY13*-activated line was a mixture isolated from eight leaves of four plants of a T_2 _family (D11UM7-2), and the RNA sample for the wild-type control was a mixture isolated from eight leaves of four Mudanjiang 8 plants. The RNA samples for *OsWRKY13*-suppressed plants were a mixture isolated from 4–6 leaves each plant at booting stage, and the RNA sample for the wild-type control was a mixture isolated from six leaves of three Minghui 63 plants. Total RNA was treated for 30 min with DNase I (Invitrogen) to remove contaminating DNA and used for quantitative reverse transcription (qRT)-PCR analysis. The qRT-PCR was conducted as described previously [32]. PCR primers for qRT-PCR are listed in Additional file 1. The expression level of actin gene was used to standardize the RNA sample for each qRT-PCR. Each qRT-PCR assay was repeated at least twice, with each repetition having three replicates; similar results were obtained in repeated experiments.

### Yeast one-hybrid assay

The interaction of OsWRKY13 protein with the DNA regulatory element was assayed by yeast one-hybrid assay according to the manufacturer's protocol (Clontech Yeast Protocols Handbook, BD Biosciences Clontech, Mountain View, CA, USA). In brief, the full-length cDNA of OsWRKY13 was obtained by RT-PCR using primers WRKY16F (5'-AT**GAATTC**GGAGTGGTGGTGGTGATG-3') harbouring a digestion site of enzyme *Eco*RI (in bold) and WRKY13R (5'-ATA**GGATCC**AGGAGCACGGCGCGGTGGC-3') harbouring a digestion site of enzyme *Bam*HI (in bold). The PCR product was ligated into the *Eco*RI/*Bam*HI cloning site of vector pGADT7-Rec2 containing a GAL4 activation domain. The target *cis*-acting DNA fragments harbouring W-box or W-box like elements were obtained by PCR amplification of the promoter regions of a series of genes using promoter-specific primers (Additional file 2). The PCR products were ligated into the *Eco*RI, *Sac*I, or *Eco*RI/*Sac*I cloning site of vector pHIS2. The negative control DNA fragment (W17, Additional file 2) without a W-box from the promoter region of *OsWRKY13 *was ligated into the *Eco*RI/*Sac*I cloning site of vector pHIS2. The yeast strain Y187 was cotransformed with pGADT7-Rec2/OsWRKY13 and pHIS2/target promoter or pHIS2/control. Positive interactions were verified by growing yeast cells on SD-Leu-Trp-His agar medium.

## Results

### A group of transcription factors was influenced by OsWRKY13

Analysis of the rice whole genome microarray data, generated using Affymetrix GeneChip Rice Genome Arrays [26], indicated that 32 transcription factor genes were differentially expressed after activation of *OsWRKY13 *(Additional file 3). Twenty-four (75%) of the differentially expressed genes were downregulated and eight (25%) of them were upregulated. Sixteen of these transcription factor genes belong to AP2/EREBP (seven), Myb (seven), and MADS (two) type transcription factors, which generally relate to the regulation of plant growth and development [33]. All of AP2/EREBP type genes were downregulated in *OsWRKY13*-activated lines. These genes appear to be involved in JA-mediated signalling pathways and/or the terpenoid metabolism pathway [26]. Furthermore, six of the seven Myb type genes and one of the two MADS type genes were also downregulated in *OsWRKY13*-activated plants. In addition, three of the four NAC type (NAM, ATAF, and CUC) genes and the two WRKY type genes were downregulated (Additional file 3). Among the downregulated NAC type genes, *SNAC1*, which is involved in abiotic stress responses [34], was also negatively regulated by OsWRKY13 during pathogen-induced defence responses [26]. The transcription factor gene with the greatest expressional change, *Os08g44830*, is putatively connected to OsWRKY13 within the flavonoid biosynthesis pathway [26]. Thus, OsWRKY13 influences the expression of a subset of genes that control some key physiological processes via interaction with W-box or W-box like *cis*-elements [9,26]. OsWRKY13 may have further effects on additional genes through other transcription factors.

### W-boxes overrepresented in the promoter regions of OsWRKY13-upregulated genes

Functional *cis*-elements on plant promoters are typically found within a 2-kb range upstream of the translation start site [18,35]. To predict the genes that are directly regulated by WRKY proteins, promoter sequences comprising the 2 kb upstream of the translation start site (ATG) were analyzed. Our previous study identified 236 upregulated and 273 downregulated genes in *OsWRKY13*-activated lines [26]. Only the promoter regions of 211 upregulated and 257 downregulated genes had transcription unit information annotated by TIGR, however, and were analyzed in this study. Using the method applied in this study to find conserved sequences on both strands of these promoters, a wide distribution of W-boxes [TTGAC, TTGAC(C/T), TTTGAC(C/T), and TTGACA] in both up- and downregulated genes was identified, but the TTGAC, TTGAC(C/T) and TTGACT elements were overrepresented in 207, 190 and 149 upregulated genes, respectively (Table 1, [19,21,36-44]). Furthermore, two conserved motifs, GTTGAC(C/T) (*P *= 4.68e-06) and TTGACCTC, were significantly enriched in both strands of the promoters of upregulated genes (Table 2, [18,19,21,22,45-55]). The two motifs contain the typical W-box TTGAC(C/T) [18,19,21]. Thus, they were considered as variant W-boxes. The GTTGACC (*P *= 1.20e-06) was more enriched than GTTGACT (*P *= 9.05e-06) in both strands of the promoters. The GTTGAC motif containing the core of a W-box (TTGAC) was also enriched in both strands of the upregulated gene promoters. These results suggest that WRKY transcription factor(s) may play important roles in the regulation of the differentially expressed genes, especially the upregulated genes in *OsWRKY13*-activated lines, but it is unknown whether these upregulated genes are directly monitored by OsWRKY13 and/or other WRKY proteins.

**Table 1:** Frequency of occurrence of known *cis*-elements in OsWRKY13-regulated genes^a^

*Cis*-Element type	Type of transcription factor	Motif sequence	Observed occurrence^b^			Expected occurrence			Reference
				
			Up	Down	Total	Up	Down	Total	
W-box (core)	WRKY	TTGAC	922*	999	1921*	780	950	1729	21
W-box	WRKY	TTGAC(C/T)	509*	529	1038*	409	498	907	21
W-box	WRKY	TTGACC	211	243	454	177	216	393	21
W-box	WRKY	TTGACT	298*	287	585*	232	283	515	21
W-box	WRKY	TTTGAC(C/T)	194	238	432	181	220	401	19
W-box	WRKY	TTGACA	269	303	572	249	304	553	21
G-box	bZIP, GBF, bHLH	CACGTG	89	113	202	90	109	199	36
as-1	bZIP, TGA-type	TGACG	498	637	1135	482	587	1069	37
DRE	AP2/EREBP, DREB	ACCGACA	26	25	51	30	36	66	38
CRT	AP2/EREBP, CBF	(A/G)CCGAC	270	290	560	275	336	611	39
GCC-box	AP2/EREBP, AP2	GCCGCC	543*	461*	1004*	623	759	1383	40
MADS	MADS	CC(A/T)6CG	30	29	59	27	33	61	41
NACRS	NAC	CATGTG	287	378	665	294	358	651	42
Myb1	MYB	(A/C)TCC(A/T)ACC	58	72	130	62	75	137	37
Myb2	MYB	TAAC(G/C)GTT	13	18	31	13	16	28	37
Myb3	MYB	TAACTAAC	19	35*	54*	13	16	29	37
Myb4	MYB	A(A/C)C(A/T)A(A/C)C	511	659	1170	515	627	1142	37
EIN3/EIL	EIN3/EIL	GGATGTA	39	33	72	39	47	85	43
CCAATBOX1	heat shock element	CCAAT	858	1015	1873	858	1045	1903	44

^a^Only the *cis*-elements putatively bound by the transcription factor types or related ones (Additional file 3), whose expression was regulated by OsWRKY13, were analyzed.

^b^*P*-values < 0.05 (chi-square test and corrected for multiple comparisons using the Bonferroni correction) in each category are indicated with an asterisk.

**Table 2:** Enumerative selection of overrepresented motifs harbouring known *cis*-elements in the promoters of OsWRKY13-regulated genes

Overrepresented motif sequence^a^	Strand^b^	*P*-value^c^	Known homologous *cis*-element			
			
			Element^d^	Transcription factor type	Potential signalling pathway	Reference
Upregulated						
G**TTGACC**	bs	1.20e-06	W-box	WRKY	biotic/abiotic response, development	18, 19, 21
G**TTGACT**	bs	9.05e-06	W-box	WRKY	biotic/abiotic response, development	18, 19, 21
G**TTGAC**	bs	1.21e-06	W-box (core)	WRKY	biotic/abiotic response, development	18, 19, 21
**TTGACC**TC	bs	3.80e-06	W-box	WRKY	biotic/abiotic response, development	18, 19, 21
T**GCTGCCG**C	ss	7.52e-06	PRE2	Rad51-like	biotic response	22
ATG**GTGA**A	ss/bs	4.99e-06	GTGA motif	unknown	pollen development	45
GTGC**AGAAA**T	ss	3.92e-06	POLLEN1LELAT52	unknown	pollen development	46
ATTC**TGTCA**G	bs	6.82e-06	BIHD1OS	homeodomain	defence response	47
						
Downregulated						
C**GTAC**G	bs/ss	2.13e-06	CURECORECR	SBP domain family	copper response	48
G**TACGTA**C	bs/ss	3.05e-06	ACGTATERD1, ACGTABOX	AP2/EREBP, bZIP	dehydration response, seed development	49, 50
A(C/G)A**GTGA**C	bs/ss	3.81e-06	GTGA motif	unknown	pollen development	45
G**AAAG**TCCGG	bs/ss	6.06e-06	DOFCOREZM/EECCRCAH1 (-)	ZF-DOF	carbon metabolism	51
G**GTTAGTT**A	bs	7.19e-07	Myb1	MYB	defence response	40
TA**TTGGTT**GT	ss	2.91e-06	REALPHALGLHCB21 (-), AREOSREP1	unknown	Phytochrome regulation gibberellin response	52, 53
C**TACT**GGC	bs	4.27e-06	CACTFTPPCA1	unknown	carbon metabolism	54
GTG**CAAT**TAT	ss	6.69e-06	CAATBOX1	unknown	tissue-specific response	55

^a ^The known *cis*-elements in the overrepresented sequences are in bold.

^b ^The letters "bs" or "ss" designate whether the element was detected as overrepresented on both strands (bs) or just on the sense strand (ss); "bs/ss" refers to consensus sequence from bs and ss with priority on both strands and "ss/bs" with priority on the sense strand.

^c ^The *P*-value of motif with bs/ss or ss/bs annotation was calculated by average of the *P*-values for bb and ss.

^d ^Dash indicates that the complementary sequence of the known *cis*-element is harboured by the conserved motif.

### A subset of WRKY family members were influenced by OsWRKY13

To examine whether the other rice WRKY family members are directly monitored by WRKY proteins, the expression profiling of WRKY family members in *OsWRKY13*-activated lines was analyzed using the microarray data (GEO database accession number GSE8380). In total, 98 WRKY family members in rice were identified from the TIGR database and the literature [56]. Analysis of the promoters of these WRKY genes showed overrepresentation of different W-boxes (*P *< 0.05, Additional file 4), suggesting that self-regulation by the WRKY family plays an important role. However, only 42 WRKY members, including overexpressed *OsWRKY13 *and downregulated *OsWRKY14 *and *OsWRKY42 *(Additional file 3), produced a hybridization signal (*P *< 0.05) in the rice whole genome microarray chip. The 42 WRKY genes were classified into two groups based on a comparison of their expression patterns in two *OsWRKY13*-activated lines and wild type (Additional file 5). Twenty-seven of the 42 WRKY genes were clustered into the downregulated group and 15 into the upregulated group, although most of the genes were not significantly differentially expressed in the chip based on the 2-fold change threshold.

**Figure 1:**
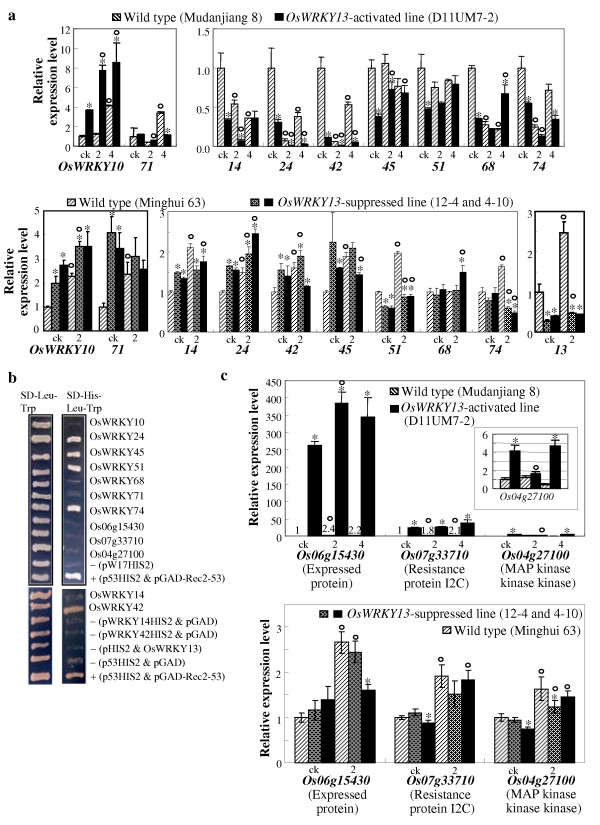
**Analyses of rice WRKY gene expression and OsWRKY13 DNA-binding activity**. (a) and (c). Expression patterns of WRKY and OsWRKY13-activated genes genes after inoculation of *Xoo *strain PXO61 at booting stage. Samples were obtained before (ck) and at 2 and 4 d after pathogen inoculation. The expression level of each gene in transgenic plants was calculated relative to that in non-inoculated wild-type plants. Circle indicates a significant difference (*P *< 0.05) between non-inoculated and inoculated plants and asterisk indicates a significant difference (*P *< 0.05) between the transgenic plant and corresponding wild type within the same treatment. Bars represent mean (three replicates) ± standard deviation. (b) Yeast one-hybrid assay using OsWRKY13 as target protein and target DNA fragments from the promoters of rice WRKY genes and three other genes as baits. +, positive control; -, negative control; pW17HIS2, *OsWRKY13 *promoter fragment without W-box. All experiments were performed twice with similar results.

Consistent with the classification in the microarray data (Additional file 5), using qRT-PCR analyses we confirmed that other WRKY genes also showed differential expression after activation of OsWRKY13 when free of pathogen infection. These include the upregulation of *OsWRKY10 *and the downregulation of *OsWRKY14*, *OsWRKY24*, *OsWRKY42*, *OsWRKY45*, *OsWRKY51*, *OsWRKY68*, and *OsWRKY74 *(Figure 1a). The analyses also showed that the expression levels of *OsWRKY10 *and *OsWRKY68 *in *OsWRKY13*-activated plants were significantly higher than that in wild type and the expression levels of *OsWRKY14*, *OsWRKY24*, *OsWRKY42*, *OsWRKY45*, and *OsWRKY71 *in *OsWRKY13*-activated plants were significantly lower than that in wild type on at least one time point after pathogen infection. Furthermore, pathogen infection significantly induced the expression of *OsWRKY10 *and *OsWRKY71 *and suppressed the expression of *OsWRKY14*, *OsWRKY24*, *OsWRKY42*, *OsWRKY68*, and *OsWRKY74 *in wild-type plants; pathogen infection also significantly induced *OsWRKY10*, *OsWRKY45*, and *OsWRKY68 *and suppressed *OsWRKY71*, *OsWRKY14*, *OsWRKY24*, *OsWRKY42*, and *OsWRKY74 *in *OsWRKY13*-activated plants (Figure 1a).

**Figure 2:**
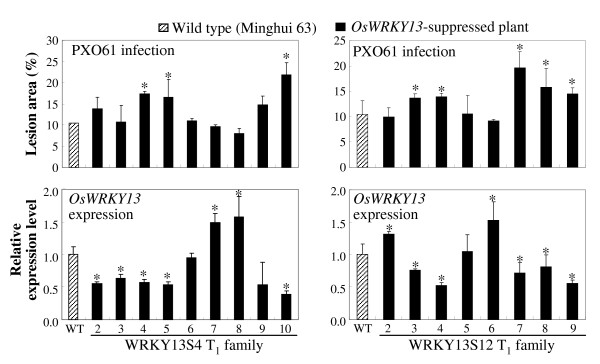
**The increased susceptibility cosegregated with suppressed expression of *OsWRKY13 *in two *OsWRKY13*-suppressed T_1 _families**. Disease was scored at 14 d after infection of *Xoo *strain PXO61. RNA samples were obtained after disease scoring. The expression level of *OsWRKY13 *in *OsWRKY13*-suppressed plants was calculated relative to that in wild-type (WT) Minghui 63. Bars represent mean (three leaves for lesion area and three replicates for expression level) ± standard deviation. Asterisk indicates a significant difference (*P *< 0.05) from wild-type Minghui 63.

To examine whether the differential expression of these WRKY genes was due to the non-physiologic overexpression of *OsWRKY13*, RNAi strategy was used to generate Os*WRKY13*-suppressed plants. Twenty-one independent transformants were obtained. These plants were inoculated with *Xoo *strain PXO61 at booting stage. Ten plants showed significantly increased susceptibility (*P *< 0.05) compared to wild-type Minghui 63 (data not shown). Four T_1 _families from four T_0 _plants, WRKY13S2, WRKY13S4, WRKY13S5, and WRKY13S12 that showed increased susceptibility or suppressed *OsWRKY13 *expression, were further analyzed for their resistance to PXO61 and *OsWRKY13 *transcript level. The increased susceptibility cosegregated with the reduced *OsWRKY13 *transcripts in the four families (Figure 2 for two families and Additional file 6 for another two families). Two independent *OsWRKY13*-suppressed T_1 _plants (WRKY13S4-10 and WRKY13S12-4), which showed increased susceptibility and suppressed *OsWRKY13 *expression, were used to analyze the expression of these WRKY genes after pathogen infection. The expression patterns of *OsWRKY71*, *OsWRKY14*, *OsWRKY24*, *OsWRKY42*, *OsWRKY45*, *OsWRKY68*, and *OsWRKY74 *in *OsWRKY13*-suppressed lines were complementary to those in *OsWRKY13*-activated plants in at least one time point examined (Figure 1b). Suppression of *OsWRKY13 *also influenced the expression of *OsWRKY10 *and *OsWRKY51*. However, the expression patterns of *OsWRKY10 *and *OsWRKY51 *in *OsWRKY13*-suppressed lines were similar as those in *OsWRKY13*-activated plants (Figure 1a). These results suggest that these WRKY genes regulated directly or indirectly by OsWRKY13 may be also involved in pathogen-induced defence responses and *OsWRKY10 *and *OsWRKY51 *may be also regulated by other transcription factor(s) that was influenced by OsWRKY13.

The nine WRKY genes analyzed (Figure 1a) all harbour W-boxes in their promoters. To evaluate whether these WRKY genes were directly influenced by OsWRKY13, yeast one-hybrid assays were performed. Detection of protein-DNA binding activity by growth performance on SD-His-Leu-Trp agar medium showed that OsWRKY13 possessed specific DNA-binding ability to the promoters of *OsWRKY24*, *OsWRKY42*, *OsWRKY45*, *OsWRKY51*, and *OsWRKY74*, but not to those of *OsWRKY10*, *OsWRKY14*, *OsWRKY68*, and *OsWRKY71 *(Figure 1b). The expression of all the genes whose promoters were bound by OsWRKY13 was suppressed in *OsWRKY13*-activated plants, suggesting that OsWRKY13 may bind preferentially to the promoters of downregulated genes *in vitro*. To examine this hypothesis, we randomly analyzed OsWRKY13 binding activity to the promoters of *Os06g15430*, *Os07g33710*, and *Os04g27100*, which showed markedly induced expression in *OsWRKY13*-activated plants and a tendency of reduced expression in *OsWRKY13*-suppressed lines (Figure 1c; [26]) and their promoters also harbour W-boxes. Yeast one-hybrid assay showed that OsWRKY13 did not bind to the promoters of the three genes (Figure 1b). Thus, OsWRKY13 appears to bind preferentially to the promoters of those genes whose expression was suppressed in *OsWRKY13*-activated plants.

### The promoters of OsWRKY13-influenced genes contain multiple types of other known cis-elements

In addition to W-boxes, other *cis*-elements required for binding of different types of transcription factors (including some of the types listed in Additional file 3: bHLH, AP2/EREBP, MADS, NAC, MYB, EIL, and CCAAT-binding protein) were identified in OsWRKY13-influenced genes (Table 1). Among these *cis*-elements, Myb3 for binding of MYB type transcription factors was overrepresented in the promoters of downregulated genes. GCC-box for binding of AP2/EREBP type transcription factors was underrepresented from both up- and downregulated genes (Table 1).

Conserved motifs harbouring known *cis*-elements were also identified in the promoters of OsWRKY13-influenced genes, but only a few of the known *cis*-elements are putatively bound by the types of transcription factors regulated by OsWRKY13 (Table 2). The GGTTAGTTA element enriched in the promoters of OsWRKY13-downregulated genes harboured the Myb1 element (GTTAGTT, [40]), putatively for MYB protein binding. The GTACGTAC motif, harbouring the ACGTATERD1 and ACGTABOX elements for binding of AP2/EREBP or bZIP types of proteins, was also enriched in OsWRKY13-downregulated genes. The other conserved motifs harbour known *cis*-elements, which are involved in biotic/abiotic responses, pollen development, and hormone responses and bound by proteins not classified among the transcription factors listed in Additional file 3 or by unknown proteins (Table 2).

### The OsWRKY13-influenced genes are enriched with novel elements in their promoters

Twelve novel elements, which were not included in the PLACE and PlantCARE databases or reported in the literature, were overrepresented in the promoters of OsWRKY13-influenced genes (Table 3). Seven of the 12 elements were located in both strands of the promoters, and the remaining five elements were strand-dependent. Novel elements 6 and 7, enriched in the promoters of OsWRKY13-downregulated genes, each comprise two four-nucleotide repeats, CGAT and AGCT, respectively. Novel element 8 (TATATATA), overrepresented in the promoters of downregulated genes, is similar to a TATA-box (C**TATA**AATAC) in rice [57]. These results suggest that the OsWRKY13-regulated genes also may be monitored by WRKY or other types of transcription factors through novel *cis*-elements.

**Table 3:** Enumerative selection of novel motifs overrepresented in the promoters of OsWRKY13-regulated genes

Gene cluster	Motif	Consensus sequence	Strand^a^	*P*-value^b^
Upregulated	novel 1	TCTCGGGCAA	ss	4.07e-06
	novel 2	GCACGGCA	bs	4.51e-06
	novel 3	ACAGGACTTA	bs	5.14e-06
	novel 4	CTATTTCGCA	ss	6.31e-06
	novel 5	GCTTGCGA	ss	8.33e-06
				
Downregulated	novel 6	CGATCGAT	ss/bs	1.40e-06
	novel 7	CAGCTAGCT	bs/ss	2.65e-06
	novel 8	TATATATA	bs/ss	4.31e-06
	novel 9	TGTGTGTGGTT	bs/ss	6.17e-06
	novel 10	TGCTTTT	ss	1.71e-06
	novel 11	TGGCCTAGAA	bs	5.35e-06
	novel 12	ACATGCCTG	ss	8.58e-06

^a^The letters "bs" or "ss" designate whether an element was detected as overrepresented on both strands (bs) or on the sense strand (ss); "bs/ss" refers to consensus sequence from bs and ss with priority on both strands and "ss/bs" with priority on the sense strand.

^b^The *P*-value of motif with bs/ss or ss/bs annotation was calculated by average of the *P*-values for bb and ss.

## Discussion

Although OsWRKY13 is potentially involved in multiple physiological processes, including disease resistance, redox homeostasis, abiotic stress responses, and development [9,26], the signalling pathways related to these processes remain to be elucidated. Our present exploration of known and putative *cis*-acting elements involved in transcriptional regulation provides a better understanding of the signal transduction from OsWRKY13 to its downstream genes.

### OsWRKY13-mediated signalling pathways are partitioned by different transcription factors

The overrepresentation of W-boxes in the promoters of upregulated genes in OsWRKY13-activated plants suggests that WRKY proteins may play important roles in the regulation of this cluster of genes. The evidence that at least nine WRKY genes are influenced by OsWRKY13 supports this hypothesis. However, the expression of eight of the nine WRKY genes was suppressed after activation of OsWRKY13 with or without pathogen infection, suggesting that some of the WRKY proteins might be expressional inhibitors of the upregulated genes in *OsWRKY13*-activated plants. The expression of all the nine WRKY genes influenced by OsWRKY13 was pathogen-responsive in *OsWRKY13*-activated, *OsWRKY13*-suppressed, and/or wild-type plants, indicating that they are also involved in host-pathogen interactions. The present results also suggest that OsWRKY13-mediated signalling pathways may be directly partitioned by some WRKY proteins, such as OsWRKY24, OsWRKY45, OsWRKY51, and OsWRKY74, whose promoters could be bound by and expression influenced by OsWRKY13. *OsWRKY24*, *OsWRKY45*, *OsWRKY51*, and *OsWRKY74 *appeared to be involved in defence pathways, because their expression was pathogen-responsive in at least one of the two wild-type plants and overexpressing *OsWRKY45 *enhances rice resistance to fungal blast [10]. Overexpressing *OsWRKY71 *enhances rice resistance to bacterial blight [8]. However, the expression of *OsWRKY45 *and *OsWRKY71 *was suppressed by OsWRKY13, an activator of disease resistance, suggesting that OsWRKY45 and OsWRKY71 may play roles other than biotic responses when OsWRKY13 is activated. This hypothesis is supported by the evidence that OsWRKY45 and OsWRKY24 repress abscisic acid (ABA) induction of the ABA-inducible *HVA22 *promoter [56]. OsWRKY51 interacts with OsWRKY71 and results in enhanced binding affinity of OsWRKY71 to the promoter of the alpha-amylase gene and suppressed expression of the gene [13].

Consistent with suppressed expression of a subset of AP2/EREBP and MYB types of transcription factors, the promoters of the downregulated genes in OsWRKY13-activated plants are enriched with elements harbouring ACGTATERD1, Myb1, and Myb3 *cis*-elements for putative binding of AP2/EREBP and MYB types of proteins. The ACGTATERD1 element is water-stress responsive [49]. Myb1 and Myb3 elements are enriched in the promoters of cold- and pathogen-inducible genes [37,40]. Activation of OsWRKY13 results in plants being more sensitive to abiotic stresses, including dehydration and cold stresses, in addition to exhibiting enhanced disease resistance [26]. Thus, the AP2/EREBP and MYB types of transcription factors may play important roles in directly monitoring the expression of OsWRKY13-downregulated genes.

### A group of novel and variant known cis-acting elements appear to be involved in OsWRKY13-mediated transcriptional regulation

OsWRKY13 and *Arabidopsis *AtWRKY70 are functional homologues in pathogen-induced defence responses, as each serves as a node of the antagonistic crosstalk between SA- and JA-dependent pathways [5,9]. However, the transcriptional regulatory mechanisms mediated by the two WRKY proteins differ. The present results show that W-boxes are only enriched in the promoters of upregulated gene in OsWRKY13-activated plants, but both up- and downregulated genes by AtWRKY70 are enriched with W-boxes [20]. The W-box like TTGAC(A/C)A and TTGAC(A/C)(C/G/T) motifs are mostly enriched in the promoters of down- and upregulated clusters by AtWRKY70, respectively [20]. The promoters of the upregulated genes by OsWRKY13 are mostly enriched with G**TTGAC(C/T) **and **TTGACC**TC motifs that harbours the typical W-box (in bold). The W-box consensus alone is insufficient for the binding of WRKY proteins and additional neighbouring nucleotides or space between adjacent W-box elements also contribute to determining high-affinity binding *in vitro *[58]. Thus, it appears that the 5'-residue G in the consensus GTTGAC(C/T) motif and 3'-residues TC in the TTGACCTC motif may be related to specific or high-affinity binding of certain WRKY protein(s) to the promoters of OsWRKY13-influenced genes. Ciolkowski *et al. *[58] reported that *Arabidopsis *AtWRKY6 and AtWRKY11 bind well to W-boxes that have a G residue directly 5' adjacent to the element, whereas AtWRKY26, AtWRKY38, and AtWRKY43 bind to the same motif if the 5'-residue is a T, C, or A. Furthermore, bacterial challenge changed the binding intensity of proteins to W-boxes [9]. Therefore, WRKY proteins may regulate the expression of the downstream genes by pathogen-induced modification such as phosphorylation or binding to diversified W-boxes.

The variant PRE2, ACGTATERD1, and Myb1 *cis*-elements for putative binding of Rad51-like, AP2/EREBP, and MYB proteins, respectively, also may be related to binding of specific proteins or function status-modified proteins. Due to the limited knowledge of *cis*-acting elements, the roles of the 12 novel conserved motifs identified in the promoter regions of OsWRKY13-influenced genes remains to be elucidated. However, overrepresentation of these motifs in the promoters of OsWRKY13-targeted genes suggests that they may play roles in OsWRKY13-mediated transcriptional regulation.

### OsWRKY13 might bind preferentially to the promoters of downregulated genes

The bindings of OsWRKY13 to the W-box-containing promoters of 18 OsWRKY13-influenced genes, including eight up- and 10 downregulated genes, have been examined *in vitro*. The present results showed that OsWRKY13 bound to the promoters of five of the eight downregulated genes examined, but could not bind to the promoters of any of the four upregulated genes examined (Figure 1b). Our previous study showed that OsWRKY13 bound specifically to the promoters of two downregulated genes, *OsAOS2 *and *OsLOX*, involved in JA synthesis in defence response and one upregulated gene, *PR1a*, functioning in SA-dependent pathway, but OsWRKY13 could not bind to the promoters of three upregulated defence-responsive genes, *OsICS1*, *NH1*, and *OsPAD4 *[9]. Furthermore, OsWRKY13 can bind to its own promoter, as revealed by gel mobility shift assays [9,22]. Self-regulation of WRKY genes by their own proteins has been reported in both negative and positive feedback control [3,4,59]. The results suggest that OsWRKY13 may function more as a negative transcriptional regulator.

## Conclusion

As a potential important transcriptional regulator of disease resistance, redox homeostasis, abiotic stress responses, and development, OsWRKY13-mediated signalling pathways are partitioned by different transcription factors through binding to distinctly distributed *cis*-acting elements in the promoters of more 500 genes. A group of novel and variant known *cis*-acting elements may contribute to OsWRKY13-mediated transcriptional regulation. WRKY proteins appear to play important roles in the monitoring of OsWRKY13-upregulated genes and genes involved in pathogen-induced defence responses, whereas MYB and AP2/EREBP proteins may contribute most to the control of OsWRKY13-downregulated genes. As some of the results were based only on the ectopic expression of *OsWRKY13*, some of the differentially expressed genes in *OsWRKY13*-activated plants may not really function in the downstream of OsWRKY13 in physiological condition. Although the actual transcriptional activation or suppression capability of OsWRKY13 remains to be determined, the present results certainly provide large amount of information for further targeted analyses of direct signal transduction from OsWRKY13 to its putatively downstream genes.

## Authors' contributions

DQ performed microarray data, promoter, gene expression, and protein-DNA interaction analyses, and drafted the manuscript. JX generated the RNAi plants and performed cosegregating analysis, and protein-DNA interaction analyses. WX carried out promoter analysis. HC analyzed protein-DNA interaction and gene expression. XL provided biochemical and molecular analysis supports. SW contributed to data interpretation and to writing the manuscript. All authors read and approved the final manuscript.

## Supplementary Material

Additional file 1 **Primers for quantitative RT-PCR analysis**. The table lists the primers sequence used for quantitative RT-PCR analysis and related GenBank accession number of each gene. Click here for file

Additional file 2 **PCR primers for amplifying promoter fragments harbouring W-box or W-box like *cis*-elements**. The table lists the primer sequences used for yeast one-hybrid assays. Click here for file

Additional file 3 **Differentially expressed transcription factor genes in *OsWRKY13*-activated lines**. The table lists the TIGR ID, fold changes, and function annotations of differentially expressed transcription factor genes in *OsWRKY13*-activated lines. Click here for file

Additional file 4 **The statistical distribution of different W-boxes in the promoters of 98 WRKY genes**. The table lists the statistical distribution of different W-boxes in the promoters of 98 WRKY genes. Click here for file

Additional file 5 **Hierarchical clustering display of expression profile of rice WRKY family genes in *OsWRKY13*-activated lines**. The figure shows the expression profile of rice WRKY family genes in *OsWRKY13*-activated lines. (A) transgenic line D11UM1-1; (B) transgenic line D11UM7-2; M, wild-type Mudanjiang 8; 1, 2, and 3, replication 1, 2, and 3. The fold changes of expressional differences of these genes were log2 transformed, clustered using the Cluster 3.0 program, and visualized by the Treeview program (Eisen et al., 1998. Proc. Natl. Acad. Sci. USA 95:14863–14868). Vertical lines on the right side indicate the genes that were further analyzed (see Figure 1). Click here for file

Additional file 6 **The increased susceptibility cosegregated with suppressed expression of *OsWRKY13 *in two *OsWRKY13*-suppressed T_1 _families**. The figure shows the cosegregating analysis of another two *OsWRKY13*-suppressed T_1 _families. Disease was scored at 14 d after infection of *Xoo *strain PXO61. RNA samples were obtained after disease scoring. The expression level of *OsWRKY13 *in *OsWRKY13*-suppressed plants was calculated relative to that in wild-type (WT) Minghui 63. Bars represent mean (three leaves for lesion area and three replicates for expression level) ± standard deviation. Asterisk indicates a significant difference (*P *< 0.05) from wild-type Minghui 63. Click here for file
