# Supplementary material for: Exploring transcriptional signalling mediated by OsWRKY13, a potential regulator of multiple physiological processes in rice
Source: BMC Plant Biol. 2009 Jun 18;9:74. doi: 10.1186/1471-2229-9-74 (PMC3224702; doi:10.1186/1471-2229-9-74)
Supplement: Additional file 1 — Primers for quantitative RT-PCR analysis. The table lists the primers sequence used for quantitative RT-PCR analysis and related GenBank accession number of each gene. [file 1471-2229-9-74-S1.doc]

**Additional file 1.** Primers for quantitative RT-PCR analysis

| Genes | Accession  number | Forward primer (5'–3') | Reverse primer (5'–3') | Expected length (bp) |
| --- | --- | --- | --- | --- |
| *OsWRKY10* | AY341854 | TCGTCGTTCTGAACGCTGAA | AGTGCATACAAGAACCGATCGA | 100 |
| *OsWRKY24* | AK107199 | TTCTTCCAGAACTCGCTCTACTGA | ACAAGTACAAAACCCCCCTAAACTAC | 100 |
| *OsWRKY45* | AK066255 | TTCCTTGTTGATGTGTCGTCTCA | CCCCCAGCTCATAATCAAGAAC | 80 |
| *OsWRKY51* | AK100954 | GATACCCCCGGTGAAGAAGAG | GCTAGTTCTACAATGATGCAGTAAAAAAA | 120 |
| *OsWRKY68* | AK072938 | CGGCAATCAGCTCGAAGATC | GTAGTAGCCCCGTGGGTAAGG | 103 |
| *OsWRKY71* | BK005074 | GTGGTGAAAGATGGGTACCAATG | CAGGCGGGAGCAAATGAG | 100 |
| *OsWRKY74* | AK065265 | ACATAGCTGATGAACACTAGAGAAGCA | CTCATCTCTATTGACCTAGGAAGAAAGC | 120 |
| *Os06g15430* | AK102606 | CGATGACTTTCATGGCAATCA | GCTGCCTCCACGGATTTG | 100 |
| *Os07g33710* | AK106593 | CCAGACGTGGACTCATTTTCTGT | GATGCCGAAAGCAAAAGCA | 100 |
| *Os04g27100* | AL606625 | GGCACCCGAGGTTGTCAA | TGTGCAACCGAGACTCCATATATC | 100 |
| *Actin* | X15865 | TGTATGCCAGTGGTCGTACCA | CCAGCAAGGTCGAGACGAA | 120 |
