# Supplementary material for: Exploring transcriptional signalling mediated by OsWRKY13, a potential regulator of multiple physiological processes in rice
Source: BMC Plant Biol. 2009 Jun 18;9:74. doi: 10.1186/1471-2229-9-74 (PMC3224702; doi:10.1186/1471-2229-9-74)
Supplement: Additional file 2 — PCR primers for amplifying promoter fragments harbouring W-box or W-box like cis-elements. The table lists the primer sequences used for yeast one-hybrid assays. [file 1471-2229-9-74-S2.doc]

**Additional file 2.** PCR primers for amplifying promoter fragments harbouring W-box or W-box like *cis*-elements

| Genes | Forward primer (5'–3')^a^ | Reverse primer (5'-3') ^a^ | Expected length (bp) |
| --- | --- | --- | --- |
| *OsWRKY10* | ATGAATTCAGAAAATTGAGCTGTCCGAAAC ^a^ | ATCGAGCTCGACGCCGGGTACGAGTAGG ^a^ | 939 |
| *OsWRKY24* | ATGAATTC TGTGGAATTACTCCTTCTCAATCC | ATCGAGCTCATTGCTTGGACGGACAGGAG | 961 |
| *OsWRKY45* | ATCGAGCTCCTCTGCCCACTGTAAAAGCC | ATCGAGCTCGTGGAGGCAGGTCGGTATTT | 985 |
| *OsWRKY51* | ATGAATTCACCAAAAGTAAAGGAGAAACCGTA | ATCGAGCTCGAGGCTAACGTAGTCGGGGTTA | 987 |
| *OsWRKY68* | ATCGAGCTCATGAGCCATCTGGCCACTTG | ATCGAGCTCAGGTGGAAATGGGGGAGAAG | 996 |
| *OsWRKY71* | ATGAATTCCCCAAAGACCATGCATCATG | ATGAATTCCGGCGAACGATTTATCACTG | 939 |
| *OsWRKY74* | ATGAATTCGTCCTCCTCCACACTTAATTTACA | ATCGAGCTCTGAGTGGGGGAGAAGAGGAT | 979 |
| *Os06g15430* | ATGAATTCACGAAGGAGACGCAATTGAGC | ATGAATTCGAAAGCTATCGATCGGCACG | 999 |
| *Os07g33710* | ATGAATTCAAAACTCCGATAGAACTTTGAACA | ATCGAGCTCCGTCGTATTTCCCTTGATCATC | 953 |
| *Os04g27100* | ATGAATTCCGGAGGGAGTATTTCTATAATTCAT | ATCGAGCTCGTCGTCAAGAAGTCCCTTTGC | 1000 |
| W17^b^ | ATGAATTCATGCGAGTGATTCGTGAGGT^1^ | ATCGAGCTCGTTGCGTCTTGGAGGAGGAGAT^2^ | 436 |

^a^ The underlined nucleotides, GAATTC and GAGCTC, are the digestion sites of enzyme *Eco*RI and *Sac*I, respectively.

^b^ Negative control (without W-box) from the promoter region of *OsWRKY13*
