# Supplementary material for: Exploring transcriptional signalling mediated by OsWRKY13, a potential regulator of multiple physiological processes in rice
Source: BMC Plant Biol. 2009 Jun 18;9:74. doi: 10.1186/1471-2229-9-74 (PMC3224702; doi:10.1186/1471-2229-9-74)
Supplement: Additional file 3 — Differentially expressed transcription factor genes in OsWRKY13-activated lines. The table lists the TIGR ID, fold changes, and function annotations of differentially expressed transcription factor genes in OsWRKY13-activated lines. [file 1471-2229-9-74-S3.doc]

**Additional file 3**. Differentially expressed transcription factor genes in *OsWRKY13*-activated lines

| TIGR^a^ ID | Fold change^b^ | Type | Functional description^c^ |
| --- | --- | --- | --- |
| Upregulated | | | |
| *Os08g44830* | 33.5 | ZF-C2H2 | zinc finger family protein, putative transparent testa 1/TT1 |
| *Os05g50080* | 2.2 | ZF-C3H | zinc finger C-x8-C-x5-C-x3-H type family protein |
| *Os06g49880* | 2.1 | ZF-B-box | B-box zinc finger family protein |
| *Os02g36924* | 11.8 | MADS | agamous-like MADS-box protein AGL21, putative |
| *Os10g25850* | 4.9 | CCAAT | CCAAT-binding transcription factor subunit B family protein |
| *Os01g18290* | 4.0 | bHLH | helix-loop-helix DNA-binding domain containing protein |
| *Os03g55590* | 2.7 | MYB | Myb-like DNA-binding domain, SHAQKYF class family protein |
| *Os06g15690* | 2.1 | NAC | NAM1/no apical meristem, putative |
|  |  |  |  |
| Downregulated | | | |
| *Os09g35010* | –6.3 | AP2/EREBP | DREB1B, AP2 domain containing protein, expressed |
| *Os03g09170* | –3.1 | AP2/EREBP | AP2 domain containing protein |
| *Os04g52090* | –2.7 | AP2/EREBP | ERF4/AP2 domain containing protein, expressed |
| *Os01g58420* | –2.4 | AP2/EREBP | OsERF3/AP2 domain containing protein, expressed |
| *Os05g41780* | –2.3 | AP2/EREBP | AP2 domain containing protein |
| *Os02g43790* | –2.2 | AP2/EREBP | BIERF3/AP2 domain containing protein, expressed |
| *Os09g35030* | –2.2 | AP2/EREBP | DREB1A protein, putative |
| *Os07g26150* | –7.2 | MYB | RADIALIS, Myb family transcription factor, putative |
| *Os02g09480* | –3.9 | MYB | Myb-like DNA-binding domain containing protein |
| *Os04g43680* | –2.5 | MYB | Myb-related protein Myb4, putative |
| *Os05g50340* | –2.4 | MYB | RADIALIS, Myb family transcription factor, putative |
| *Os05g51160* | –2.2 | MYB | putative MCB2 protein/Myb transcription factor |
| *Os11g47460* | –2.2 | MYB | Myb family transcription factor, putative |
| *Os06g09310* | –3.1 | ZF-C3H | zinc finger, C3HC4 type family protein |
| *Os03g55540* | –2.5 | ZF-C2H2 | zinc-finger protein 1, C2H2 type family, putative |
| *Os09g21710* | –2.4 | ZF-AN1 | AN1-like zinc finger family protein |
| *Os09g38110* | –2.4 | ZF-C3H | zinc finger, C3HC4 type family protein |
| *Os08g10080* | –3.1 | NAC | NAM/NAC-domain containing protein 21/22, putative |
| *Os01g48460* | –2.9 | NAC | no apical meristem protein |
| *Os03g60080* | –2.1 | NAC | SNAC1/NAC-domain containing protein 19, expressed |
| *Os02g26430* | –4.0 | WRKY | OsWRKY42 |
| *Os01g53040* | –2.7 | WRKY | OsWRKY14 |
| *Os07g41370* | –3.0 | MADS | MADS-box transcription factor 18, putative |
| *Os08g39830* | –2.6 | EIL | ETHYLENE-INSENSITIVE3-like 3 protein, putative |

^a^TIGR, the Institute for Genomic Research.

^b^The average value of probe set(s) was used as the expression ratio of one gene.

^c^Putative function is based on Affymetrix annotation and BLAST hits against the nr database of the National Center for Biotechnology Information (http://www.ncbi.nlm.nih.gov).
