# Supplementary material for: Exploring transcriptional signalling mediated by OsWRKY13, a potential regulator of multiple physiological processes in rice
Source: BMC Plant Biol. 2009 Jun 18;9:74. doi: 10.1186/1471-2229-9-74 (PMC3224702; doi:10.1186/1471-2229-9-74)
Supplement: Additional file 4 — The statistical distribution of different W-boxes in the promoters of 98 WRKY genes. The table lists the statistical distribution of different W-boxes in the promoters of 98 WRKY genes. [file 1471-2229-9-74-S4.doc]

**Additional file 4.** The statistical distribution of different W-boxes in the promoters of 98 WRKY genes

| W-box sequence | Observed occurrence of motif ^a^ | Expected occurrence of motif | Observed occurrence of gene^b^ | Expected occurrence of gene |
| --- | --- | --- | --- | --- |
| TTGAC | 497* | 362 | 94 | 93 |
| TTGAC[CT] | 287* | 190 | 89 | 79 |
| TTGACC | 118* | 82 | 67 | 53 |
| TTGACT | 170* | 108 | 72 | 61 |
| TTTGAC[CT] | 123* | 84 | 55 | 50 |
| TTGACA | 148 | 116 | 73 | 65 |

^a^The number indicates the total number of each type W-box in the promoters of all the WRKY genes. *P-*values < 0.05 (chi-square test and corrected for multiple comparisons using the Bonferroni correction) in each category are indicated with an asterisk.

^b^The number indicates the total number of WRKY genes, which promoters contain at least one of the related W-box.
