# Supplementary material for: Exploring transcriptional signalling mediated by OsWRKY13, a potential regulator of multiple physiological processes in rice
Source: BMC Plant Biol. 2009 Jun 18;9:74. doi: 10.1186/1471-2229-9-74 (PMC3224702; doi:10.1186/1471-2229-9-74)
Supplement: Additional file 5 — Hierarchical clustering display of expression profile of rice WRKY family genes in OsWRKY13-activated lines. The figure shows the expression profile of rice WRKY family genes in OsWRKY13-activated lines. (A) transgenic line D11UM1-1; (B) transgenic line D11UM7-2; M, wild-type Mudanjiang 8; 1, 2, and 3, replication 1, 2, and 3. The fold changes of expressional differences of these genes were log2 transformed, clustered using the Cluster 3.0 program, and visualized by the Treeview program (Eisen et al., 1998. Proc. Natl. Acad. Sci. USA 95:14863–14868). Vertical lines on the right side indicate the genes that were further analyzed (see Figure 1). [file 1471-2229-9-74-S5.ppt]

## Slide 1
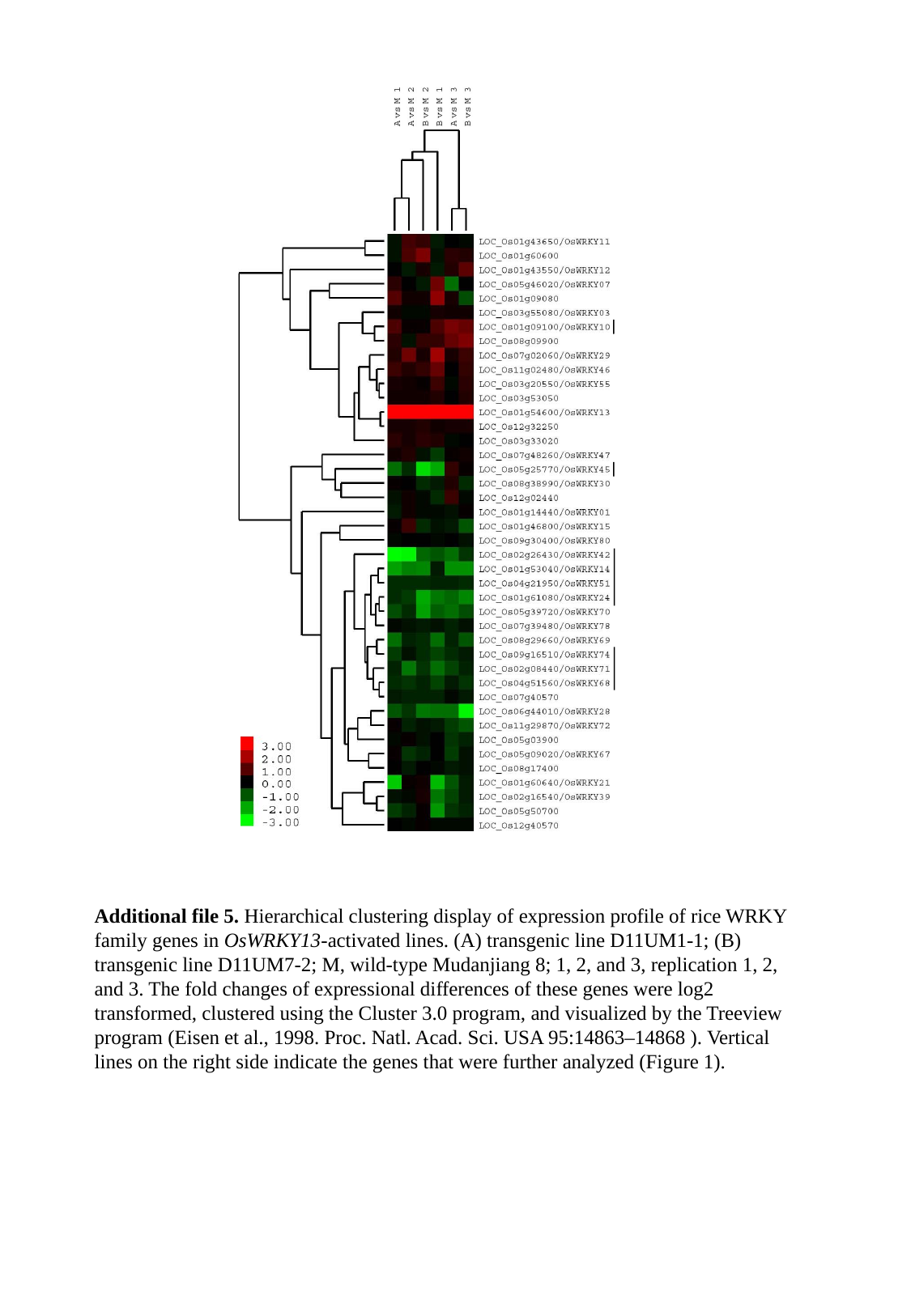

Additional file 5. Hierarchical clustering display of expression profile of rice WRKY family genes in OsWRKY13-activated lines. (A) transgenic line D11UM1-1; (B) transgenic line D11UM7-2; M, wild-type Mudanjiang 8; 1, 2, and 3, replication 1, 2, and 3. The fold changes of expressional differences of these genes were log2 transformed, clustered using the Cluster 3.0 program, and visualized by the Treeview program (Eisen et al., 1998. Proc. Natl. Acad. Sci. USA 95:14863–14868 ). Vertical lines on the right side indicate the genes that were further analyzed (Figure 1).
