# Supplementary material for: Exploring transcriptional signalling mediated by OsWRKY13, a potential regulator of multiple physiological processes in rice
Source: BMC Plant Biol. 2009 Jun 18;9:74. doi: 10.1186/1471-2229-9-74 (PMC3224702; doi:10.1186/1471-2229-9-74)
Supplement: Additional file 6 — The increased susceptibility cosegregated with suppressed expression of OsWRKY13 in two OsWRKY13-suppressed T1 families. The figure shows the cosegregating analysis of another two OsWRKY13-suppressed T1 families. Disease was scored at 14 d after infection of Xoo strain PXO61. RNA samples were obtained after disease scoring. The expression level of OsWRKY13 in OsWRKY13-suppressed plants was calculated relative to that in wild-type (WT) Minghui 63. Bars represent mean (three leaves for lesion area and three replicates for expression level) ± standard deviation. Asterisk indicates a significant difference (P < 0.05) from wild-type Minghui 63. [file 1471-2229-9-74-S6.ppt]

## Slide 1
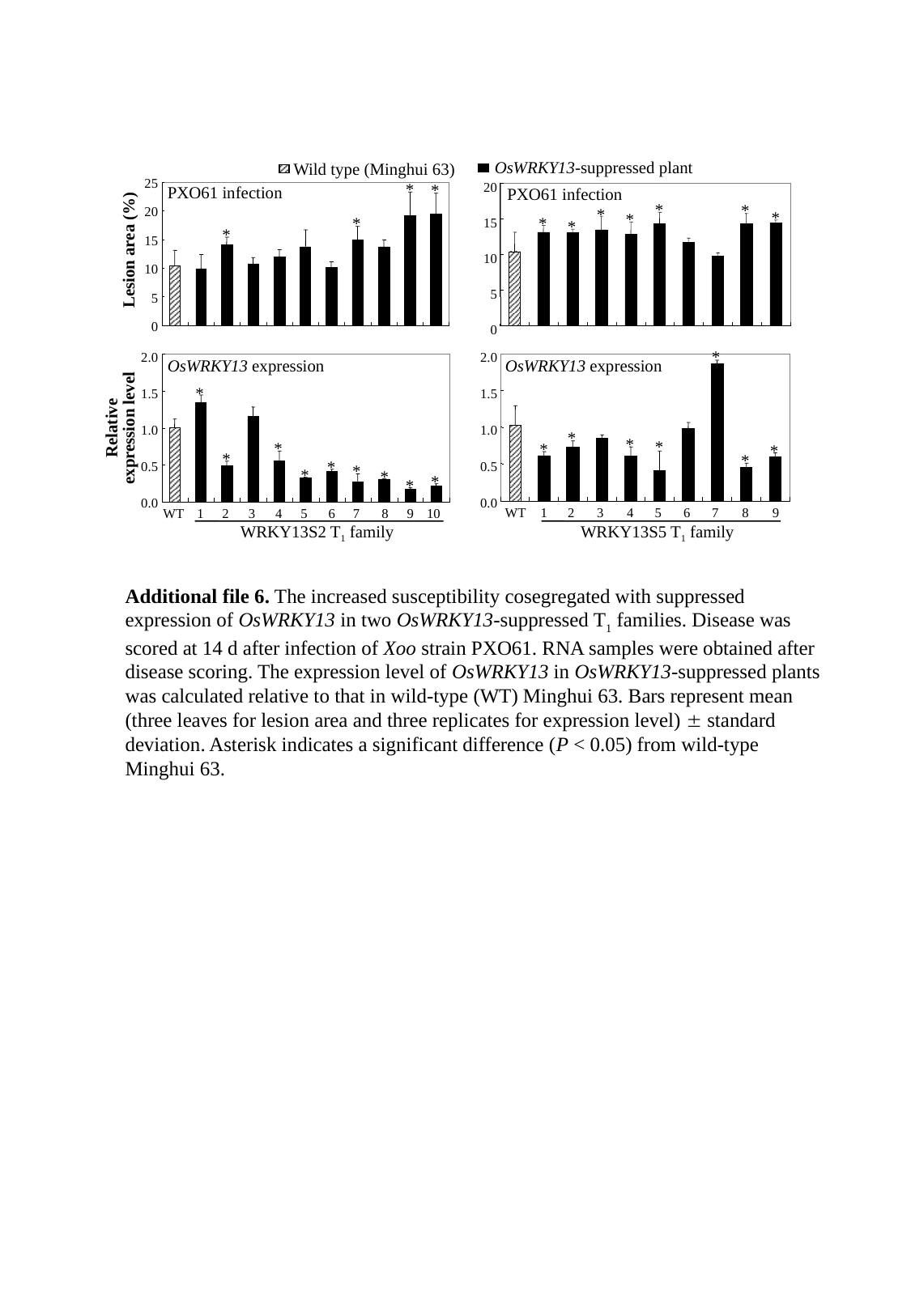

OsWRKY13-suppressed plant
Wild type (Minghui 63)
*
*
25
20
15
10
5
0
PXO61 infection
PXO61 infection
20
15
10
5
0
*
*
*
*
*
*
*
*
*
Lesion area (%)
*
2.0
1.5
1.0
0.5
0.0
OsWRKY13 expression
2.0
1.5
1.0
0.5
0.0
OsWRKY13 expression
*
Relative
expression level
*
*
*
*
*
*
*
*
*
*
*
*
*
*
WT
1
2
3
4
5
6
7
8
9
WT
1
2
3
4
5
6
7
8
9
10
WRKY13S2 T1 family
WRKY13S5 T1 family
Additional file 6. The increased susceptibility cosegregated with suppressed expression of OsWRKY13 in two OsWRKY13-suppressed T1 families. Disease was scored at 14 d after infection of Xoo strain PXO61. RNA samples were obtained after disease scoring. The expression level of OsWRKY13 in OsWRKY13-suppressed plants was calculated relative to that in wild-type (WT) Minghui 63. Bars represent mean (three leaves for lesion area and three replicates for expression level) ± standard deviation. Asterisk indicates a significant difference (P < 0.05) from wild-type Minghui 63.
